# ﻿Taxonomic diversity of amphibians (Amphibia, Anura) and reptiles (Reptilia, Testudines, Squamata) in a heterogeneous landscape in west-central Mexico: a checklist and notes on geographical distributions

**DOI:** 10.3897/zookeys.1211.122565

**Published:** 2024-09-02

**Authors:** Verónica Carolina Rosas-Espinoza, Eliza Álvarez-Grzybowska, Arquímedes Alfredo Godoy González, Ana Luisa Santiago-Pérez, Karen Elizabeth Peña-Joya, Fabián Alejandro Rodríguez-Zaragoza, Leopoldo Díaz Pérez, Francisco Martín Huerta Martínez

**Affiliations:** 1 Laboratorio de Ecología Molecular, Microbiología y Taxonomía (LEMITAX), Departamento de Ecología Aplicada,, Centro Universitario de Ciencias Biológicas y Agropecuarias, Universidad de Guadalajara, Camino Ramón Padilla Sánchez 2100, CP 45200, Zapopan, Jalisco, Mexico Universidad de Guadalajara Zapopan Mexico; 2 Departamento de Producción Forestal, Centro Universitario de Ciencias Biológicas y Agropecuarias, Universidad de Guadalajara, Camino Ramón Padilla Sánchez 2100, CP 45200, Zapopan, Jalisco, Mexico Universidad de Guadalajara Puerto Vallarta Mexico; 3 Laboratorio de Ecología, Paisaje y Sociedad, Centro Universitario de la Costa, Universidad de Guadalajara, Puerto Vallarta 48280, Jalisco, Mexico Universidad de Guadalajara Zapopan Mexico; 4 Centro de Estudios en Interacciones Ecológicas, Departamento de Ecología, Centro Universitario de Ciencias Biológicas y Agropecuarias, Universidad de Guadalajara, Camino Ramón Padilla Sánchez 2100, CP 45200, Zapopan, Jalisco, Mexico Universidad de Guadalajara Puerto Vallarta Mexico

**Keywords:** Crops, herpetofauna, Jalisco state, native vegetation, range extension

## Abstract

In Mexico, land use changes have significantly impacted the diversity of amphibians and reptiles in a negative way. In light of this, we evaluate the alpha and beta components of the taxonomic diversity of amphibians and reptiles in a heterogeneous landscape in west-central Mexico. Additionally, we provide a checklist of amphibian and reptile species recorded over nine years of observations within the studied landscape and surrounding areas. The land cover/use types with the highest species richness and alpha taxonomic diversity differed between amphibians and reptiles. Overall beta taxonomic diversity was high for both groups, but slightly higher in reptiles. This taxonomic differentiation mainly corresponded to a difference in the turnover component and was greater in pristine habitats compared to disturbed ones. The checklist records 20 species of amphibians (ten of which are endemic) and 48 of reptiles (30 endemics). Additionally, the study expands the known geographical distribution range of one species of frog and three species of snakes. Our findings suggest that heterogeneous landscapes with diverse land cover/use types can provide essential habitats for the conservation of amphibian and reptile species.

## ﻿Introduction

Amphibians and reptiles are abundant and diverse components of terrestrial and freshwater ecosystems, serving various ecological functions (Pough et al. 2004; Wells 2007). Mexico harbors 418 species of amphibians (AmphibiaWeb 2023; Ramírez-Bautista et al. 2023) and 1,044 of reptiles (Ramírez-Bautista et al. 2023; Uetz 2024), which account for 4.9% of the world’s amphibians and 8.9% of its reptiles. Besides, 65% of the amphibians and 57% of the reptiles are endemic to Mexico, occurring predominantly in the Trans-Mexican Volcanic Belt and the Balsas Depression (Ochoa-Ochoa and Flores-Villela 2006).

Human pressure on natural environments has been intensifying mainly with agricultural landscapes becoming increasingly dominant. Land-use changes threaten biodiversity, primarily through habitat loss and degradation (Fischer and Lindenmayer 2007; Sayer et al. 2013; Davison et al. 2021). Amphibians and reptiles have very distinct physiologies, biologies, and ecological traits (Pough et al. 2004; Wells 2007). As a result, they tend to differ in their responses to environmental disturbances (Koleff and Guyer 2008). Particularly amphibians are often more susceptible to these changes due to their permeable skin, and their communities can show significant shifts in taxonomic and functional diversity (Ernst et al. 2006; Ernst and Rödel 2008). In disturbed environments, amphibian and reptile communities are less diverse than in pristine or protected ones mainly due to microhabitat loss, lack of food (Gardner et al. 2007; Trimble and Aarde 2014; Thompson et al. 2015) competition, predation, spread of diseases from invasive species (Bucciarelli et al. 2014; Kraus 2015; Falaschi et al. 2020), in addition to habitat alteration and hybridization (Falaschi et al. 2020). Also, disturbed habitats can lead to significant species turnover at the landscape scale, favoring generalist or invasive species while also sustaining a few native species (Wanger et al. 2010).

One approach used to quantify the taxonomic complexity of species assemblages at the local level and to evaluate the response of organisms to spatial gradients and differentiation at the regional level has been to analyze the alpha (local) and beta (turnover) diversity of these assemblages (Baselga 2010; Jost et al. 2011).The more traditional diversity measures (e.g., species richness, Shannon index, Jaccard index, etc.) of alpha and beta diversity (often referred to as ecological) assume that all species carry the same weight (Magurran 2004; Chao et al. 2010), and thus fail to consider taxonomic diversity of species and their evolutionary past (Gaston and Spicer 2004). As a result, methods have been developed to add this dimension (Warwick and Clarke 1995). Alpha and beta diversity can be assessed by incorporating supra-specific levels associated with each species (e.g., genus, family, and order) to capture information on their phylogenetic diversity and their ecological and evolutionary histories (Warwick and Clarke 1998; Nipperess et al. 2010; Carvalho et al. 2012). This approach can even be used with large assemblages that lack species-level phylogenies (Izsak and Price 2001; Carvalho et al. 2012).

The alpha component of taxonomic diversity consists in the distinctiveness of taxa which measures the degree of taxonomic relatedness of species present in each sample, a reflection of the ecological and evolutionary mechanisms that have contributed to taxonomic composition (Warwick and Clarke 1998). Beta taxonomic diversity can be divided into turnover (replacement) and differences in richness (loss or gain) components (Baccaro et al. 2007; Carvalho et al. 2012). The measure of differences in richness is useful to understand how habitat conditions affect communities and, when assessing highly heterogeneous landscapes, the patchiness of the species that inhabit them (Melo et al. 2009; Ochoa-Ochoa et al. 2014).

Our understanding of taxonomic diversity of amphibians and reptiles in Mexico has been enriched by several studies. Cruz-Elizalde et al. (2014) showed that the richest environments for reptile species in the Chihuahuan Desert were not taxonomically diverse. Hernández-Salinas et al. (2023), who measured taxonomic and functional diversity in amphibian and reptile communities in six vegetation types in Durango, found that vegetation types that had more complex family and genera networks differed between the two groups. Díaz de la Vega-Pérez et al. (2019) recorded high amphibian and reptile dissimilarities between habitats at La Malinche National Park. Many have reported high overall values of taxonomic beta diversity for amphibians and reptiles compared to other vertebrate groups (Koleff et al. 2008; Ochoa-Ochoa et al. 2014; Calderón-Patrón et al. 2016). However, between these two groups Calderón-Patrón et al. (2016) recorded higher beta diversity (species) and beta taxonomic diversity (species and associated taxonomic levels) for reptiles and Ochoa-Ochoa et al. (2014) obtained this same result particularly for the central and northwest regions of Mexico.

Although in Mexico amphibians and reptile species have been documented as disappearing because of human activity such as habitat fragmentation, pollution, pet trade, invasive species, emerging diseases, and global warming, they are still the less well-studied vertebrate groups (Ceballos et al. 2015). In this study, we report on 1) the spatial variation of alpha (Warwick and Clarke 1998) and beta (Carvalho et al. 2012) taxonomic diversity of amphibians and reptiles in a heterogeneous landscape in west-central Mexico, and 2) a checklist including some species’ geographic range extensions. We first hypothesized that species richness and alpha taxonomic diversity of amphibians and reptiles would be highest in the same habitats across the heterogeneous landscape. Secondly, we predicted a lower species richness and taxonomic diversity in the land use types of corn and sugarcane crops for both groups and, for amphibians, higher species richness, and alpha taxonomic diversity in the riparian habitat surrounded by tropical dry forest (RH-TDF). We made these predictions because habitat complexity, the presence of permanent water (especially for amphibians), and less habitat disturbance encourage patterns of higher species richness and taxonomic distinctiveness. Thirdly, we expected beta diversity to be high for both groups due to the heterogeneity of the landscape, and for it to be higher for amphibians than reptiles with a strong turnover component due to their lower mobility as reported in previous work. Finally, we predicted higher dissimilarities between the land use types and the land cover types for both groups.

## ﻿Materials and methods

### ﻿Study area

**Figure 1.**
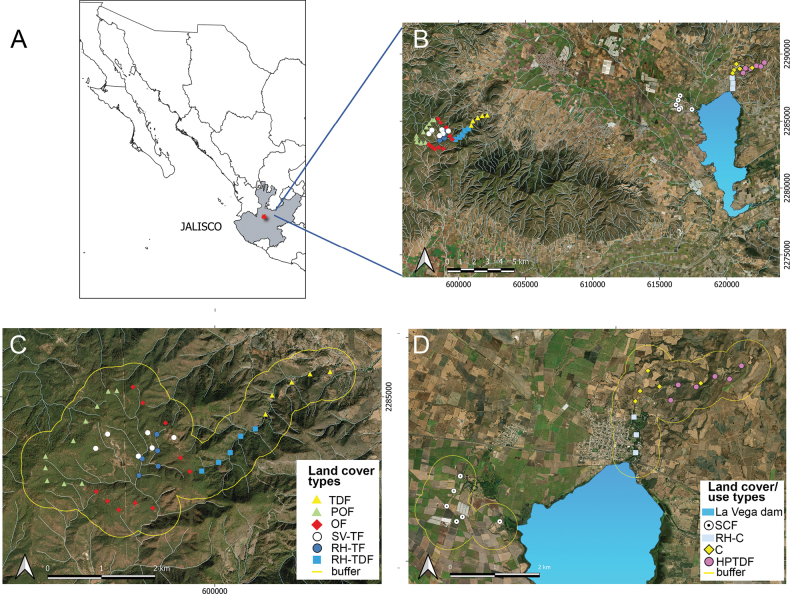
**A** study area in Jalisco, Mexico **B** sampling points in the landscape. Sampling plots (**C, D**). Codes: sugar cane field (SCF), riparian habitat surrounded by crops (RH-C), cornfield (C), highly perturbed tropical dry forest (HPTDF), tropical dry forest (TDF), riparian habitat surrounded by tropical dry forest (RH-TDF), riparian habitat surrounded temperate forest (RH-TF), secondary vegetation surrounded by temperate forest (SV-TF), oak forest (OF) and pine-oak forest (POF) (modified after Rosas-Espinoza et al. 2022).

The study area consisted of different land cover/use types within the municipalities of Ahualulco de Mercado (main population 20°42'6.84″N, 103°58'24.96″W) and Teuchitlán (20°40'59.88″N, 103°50'51.72″W), both located in the west-central state of Jalisco, México (Fig. 1). The territory of Ahualulco de Mercado ranges in elevation between 1280 and 2600 m a.s.l. The weather is semi-warm / semi-humid, with a mean annual temperature of 20.5 °C and average minimum and maximum temperatures of 7.9 °C and 33.3 °C, respectively. The average annual precipitation is 900 mm, with a cumulative average of 643.27 mm ( IIEG 2023a). In Teuchitlán, the elevation ranges between 1247 and 2392 m a.s.l. The weather is semi-dry / semi-warm, with a mean annual temperature of 21.2 °C and average maximum and minimum temperatures of 33.5 °C and 8.4 °C, respectively (IIEG 2023b). Mean annual precipitation is 948 mm, with an average accumulated precipitation of 634.56 mm; rain occurs mainly in the summer, and dry periods happen during spring and winter (IIEG 2023b). We selected the following ten main land cover/use types (sampling sites): sugar cane field (SCF), riparian habitat surrounded by crops (RH-C), cornfield (C), highly perturbated tropical dry forest (HPTDF), tropical dry forest (TDF), riparian habitat surrounded by tropical forest (RH-TDF), riparian habitat surrounded by temperate forest (RH-TF), secondary vegetation surrounded by temperate forest (SV-TF), oak forest (OF) and pine-oak forest (POF) (Fig. 1).

The municipalities of Ahualulco de Mercado and Teuchitlán have similar territorial areas of 235.25 and 211.18 square kilometers, respectively. The two neighboring municipalities share a broad valley. The predominant land use types are agricultural and livestock activity, covering 60% of its surface, followed by secondary vegetation at 17% (IIEG 2023a, 2023b). The SCF land use type was located between 1258 and 1390 m a.s.l. Both municipalities are important for sugarcane production in Jalisco (SIAP 2018). The C was found on the lower slopes of the mountains between 1250 and 1440 m a.s.l. Clearing events for agriculture have occurred in these areas since pre-Columbian times. The predominant crops are rainfed corn, *Zeamays*, and *Agavetequilana* (Santiago-Pérez 2023).

The RH-C land cover type is found between 1265 and 1270 m a.s.l. along a stretch of the Teuchitlán River. The dominant tree species were *Salixhumboldtiana*, *Fraxinusuhdei*, *Ficusinsipida*, *Lysilomaacapulcense*, *Baccharissalicifolia*, *Salixtaxifolia*, *Arundodonax* and *Scirpuscalifornicus*. One riverbank is used for crops, the other for recreational activities. HPTDF, with a high disturbance level, was found between 1200 and 1500 m a.s.l., and the dominant tree species were *Acaciafarnesiana*, *A.pennatula*, *Prosopislaevigata*, and *Pithecellobiumdulce*. The TDF was found between 1200 and 1700 m a.s.l. and included *Burserabipinnata*, *Ipomoeamurucoides*, *L.acapulcense*, *Opuntiafuliginosa*, and *Tecomastans* as dominant species (García-Martínez and Rodríguez 2018). The TDF had both temporary and permanent water bodies, but some areas were deforested, so C and SCF were established instead (Rosas-Espinoza et al. 2022). The TDF in the archaeological zone of Guachimontones (1300 to 1482 m a.s.l.) had frequent *Burserafagaroides*, *B.bipinnata*, *B.palmeri*, *Ipomoeaintrapilosa*, *Heliocarpusterebinthinaceus*, *Guazumaulmifolia*, *Eysenhardtiapolystachya*, and *Leucaenaleucocephala* (Santiago-Pérez 2023).

The RH-TDF had permanent streams. It was found between 1450 and 1750 m a.s.l. and was dominated by *L.acapulcense*, *Lippiaumbellata*, *Eysenhardtiapolystachya* and *I.intrapilosa*. The RH-TF had both permanent and temporal streams. It was located between 1500 and 1800 m a.s.l., and the dominant tree species were *Salixbonplandiana*, *Quercusmagnoliifolia*, *Q.splendens*, *Q.obtusata*, *Aioueapachypoda*, and *Oreopanaxpeltatus* (García-Martínez and Rodríguez 2018). The dominant tree species in SV-TF (1550–1750 m a.s.l.) were *I.intrapilosa*, *T.stans*, *A.farnesiana*, *Verbesinagreenmanii*, *Solanummadrense*, and *Sennafoetidissima* (García-Martínez and Rodríguez 2018). An artificial pond had water yearly (Rosas-Espinoza et al. 2022). The OF was found between 1500 and 1900 m a.s.l., with *Quercusresinosa*, *Q.magnoliifolia*, *Q.castanea*, and *Q.gentry* as the dominant tree species. The POF was found between 1800 and 2590 m a.s.l. The predominant tree species were *Q.resinosa*, *Pinusoocarpa*, *P.devoniana*, and *P.lumholtzii* (García-Martínez and Rodríguez 2018).

### ﻿Amphibian and reptile surveys for taxonomic diversity measurements

We established circular diurnal (500 m^2^ each one) and rectangular nocturnal (10,000 m^2^) survey plots in each land cover/use type. The diurnal plots were separated 400 m of distance from each one. At each plot an intensive unrestricted visual search was carried out on the microhabitats preferred by these reptile species in each point count (i.e., logs and rocks). We recorded all individuals observed, and when possible, measured and photographed them. They were later released at the capture site. We conducted nine, monthly, samplings of the amphibian and reptile communities from July 2011 to August 2012 in TDF, RH-TDF, RH-TF, SV-TF, OF, and POF. Additionally, we surveyed both groups of taxa for eight months from September 2012 to September 2013 in SCF, RH-C, C, and HPTDF. Once a month during the day, we surveyed 12,500 m^2^ in TDF, RH-TDF, and RH-TF; 15,000 m^2^ in SCF, C, and HPTDF; 17,500 m^2^ in SV-TF; 25,000 m^2^ in OF and POF; and 22,500 m^2^ in RH-C. And at night we surveyed 10,000 m^2^ in each land cover/use type.

### ﻿Amphibian and reptile checklist and distribution extensions

We included in the checklist all species whose presence within the study area or surroundings was confirmed by direct observation between August 2011 and December 2020. To corroborate a species’ identity, we took and deposited photographs in the Colección Herpetológica of the Museo de Zoología in the Facultad de Estudios Superiores Zaragoza, Universidad Autónoma de México. We followed Frost (2023) for the taxonomy of amphibians, Uetz (2024) and Zaher et al. (2019) for that of the reptiles, and CONABIO (2023) for common names. We considered various scientific sources to determine the endemism of amphibians and reptiles (e.g., Cruz-Sáenz et al. 2017; Johnson et al. 2017; Frost 2023; AmphibiaWeb 2023; Ramírez-Bautista et al. 2023). We consulted the Mexican government threatened species list NOM-059 (SEMARNAT 2019), IUCN (2024) and Environmental Vulnerability Score (EVS; Wilson et al. 2013a, 2013b) to establish each species’ conservation status. We used the species distribution maps published by the Red List of Threatened Species of the IUCN (2024) and records from Global Biodiversity Information Facility (GBIF). We measured the distance of our records to the closest observations in the region. We determined a species range extension when an observation was made at least 20 km in a straight line from the nearest record.

### ﻿Statistical analysis of the taxonomic diversity

We generated monthly matrices of presence-absence of amphibian and reptile species. We determined sampling effort in each land/use type and the whole study area using sample-based rarefaction curves using the non-parametric estimators Chao 2, Jackknife 1, and Jackknife 2. All rarefaction curves were built using 10,000 randomizations without replacement. We performed these analyses using EstimateS 9.1.0 (Colwell 2013).

### ﻿Alpha taxonomic diversity

We measured the alpha component of taxonomic diversity by computing taxonomic distinctness. It takes into consideration the degree of taxonomic relatedness among species in each sample as a reflection of the ecological and evolutionary mechanisms that contribute to taxonomic composition (Warwick and Clarke 1998). To quantify the degree of taxonomic relatedness among species in the various land covers/use types, we calculated the average taxonomic distinctness (Delta, Δ^+^) and its variation (Lambda, Λ^+^) (Warwick and Clarke 1995; Clarke et al. 2014) per land cover/use type for all amphibians and reptiles’ assemblages. We built a five-level taxonomic aggregation matrix that included species, genus, family, order, and phylum. We used the same weight for all taxonomic levels (ω = 1). We created the models with a 95% confidence interval by carrying out 10,000 permutations and with a ratio of 1.2 species (Clarke and Warwick 1998). All analyses (∆ + and Λ+) were performed using PRIMER 7.0.21 and PERMANOVA +1 (Clarke and Gorley 2015).

### ﻿Beta taxonomic diversity

We measured the beta component of taxonomic diversity by calculating and partitioned taxonomic beta into turnover (*β.3*) and differences in richness (*βrich*) components following Baccaro et al. (2007) and Carvalho et al. (2012). *Bcc* represents the total dissimilarity (1 – Jaccard’s similarity coefficient) split between the components of dissimilarity (*β.3*) and dissimilarity due to differences in richness (*βrich*). We considered four supra-specific levels (species, genus, family, and order) for both groups. We assessed taxonomic beta diversity and partitioning of components using the “BAT” package (Cardoso et al. 2024) and Carvalho et al. (2012) script. We performed these analyses using the R studio program R-project 4.1.1 (R Development Core Team 2022).

## ﻿Results

**Figure 2.**
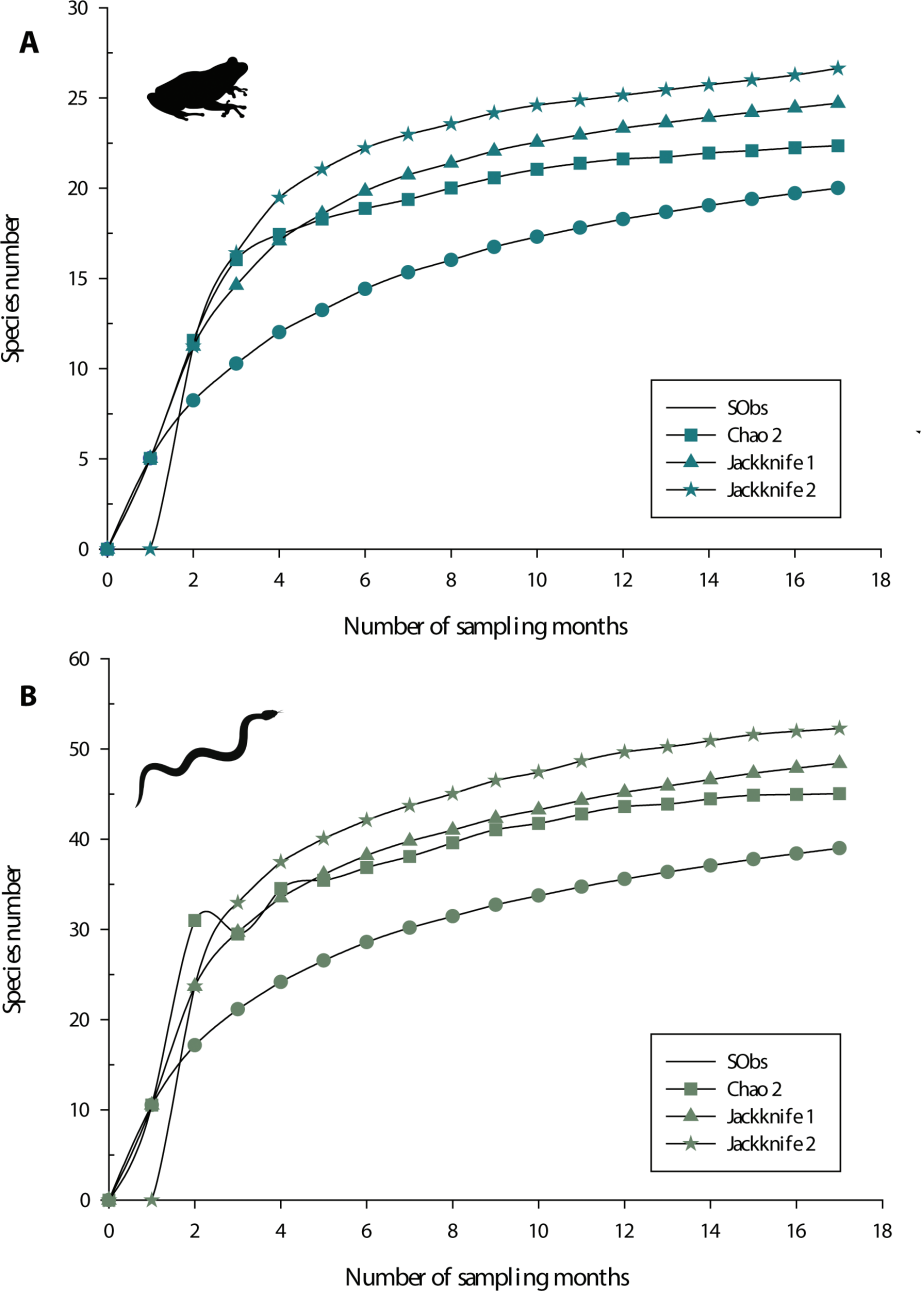
Sample-based rarefaction curves for amphibians and reptiles generated using presence-absence data, showing observed and expected species for the different land cover/use types using non-parametric estimators. Codes: sugar cane field (SCF), riparian habitat surrounded by crops (RH-C), cornfield (C), highly perturbated tropical dry forest (HPTDF), tropical dry forest (TDF), riparian habitat surrounded by tropical forest (RH-TDF), riparian habitat surrounded temperate forest (RH-TF), secondary vegetation surrounded by temperate forest (SV-TF), oak forest (OF), pine-oak forest (POF), and number of species observed (Sobs).

We recorded 20 species of amphibians and 39 of reptiles in the study area between August 2011 and September 2013. The average sampling effort for amphibians was 81.8% of representativity for the study area and that for reptiles, 80.5% (Fig. 2). The average sampling effort varied between 61% and 95.6% of representativity between all land cover/use types for amphibians, and between 65.3% and 97.6% for reptiles (Suppl.material 1: table S1).

### ﻿Alpha taxonomic diversity

Numerically, we recorded the lowest amphibian species richness in POF (three species) compared to the highest richness in HPTDF (eight), RH-C (nine), and RH-TDF (ten). Medium species richness was recorded in OF (five), TDF (five), RH-TF (six), CO (six), SCF (seven), and SV-TF (seven). In contrast, we recorded the lowest species richness for reptiles in RH-TF (five), CA (seven), C (seven), and POF (nine). SV-TF (18) had the highest richness. Medium species richness was registered in RH-TDF (10), OF (12), HPTDF (12), RH-C (13), and TDF (14).

**Figure 3.**
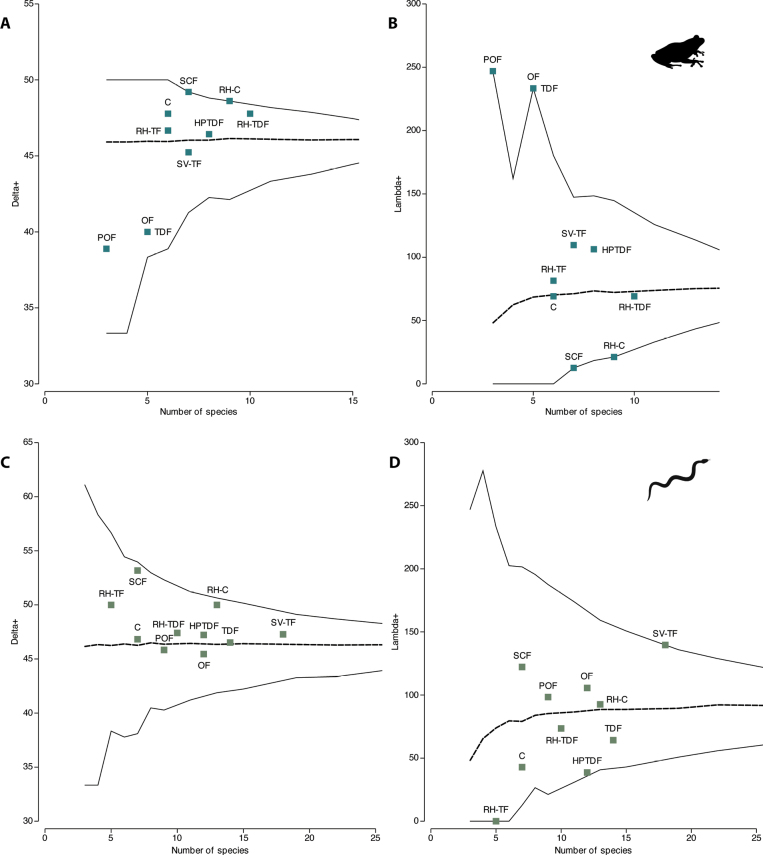
Average taxonomic distinctness (Δ+) and its variance (Λ+) by land cover/use type of **A** amphibians and **B** reptiles in a heterogeneous landscape at west-central Mexico.

The average taxonomic distinctness for amphibians and reptiles had all the ∆+ and Λ+ values within the probability funnels (p>0.05) (Fig. 3), which indicates that the alpha taxonomic richness of amphibians and reptiles was within the model’s expectations.

### ﻿Beta taxonomic diversity

**Figure 4.**
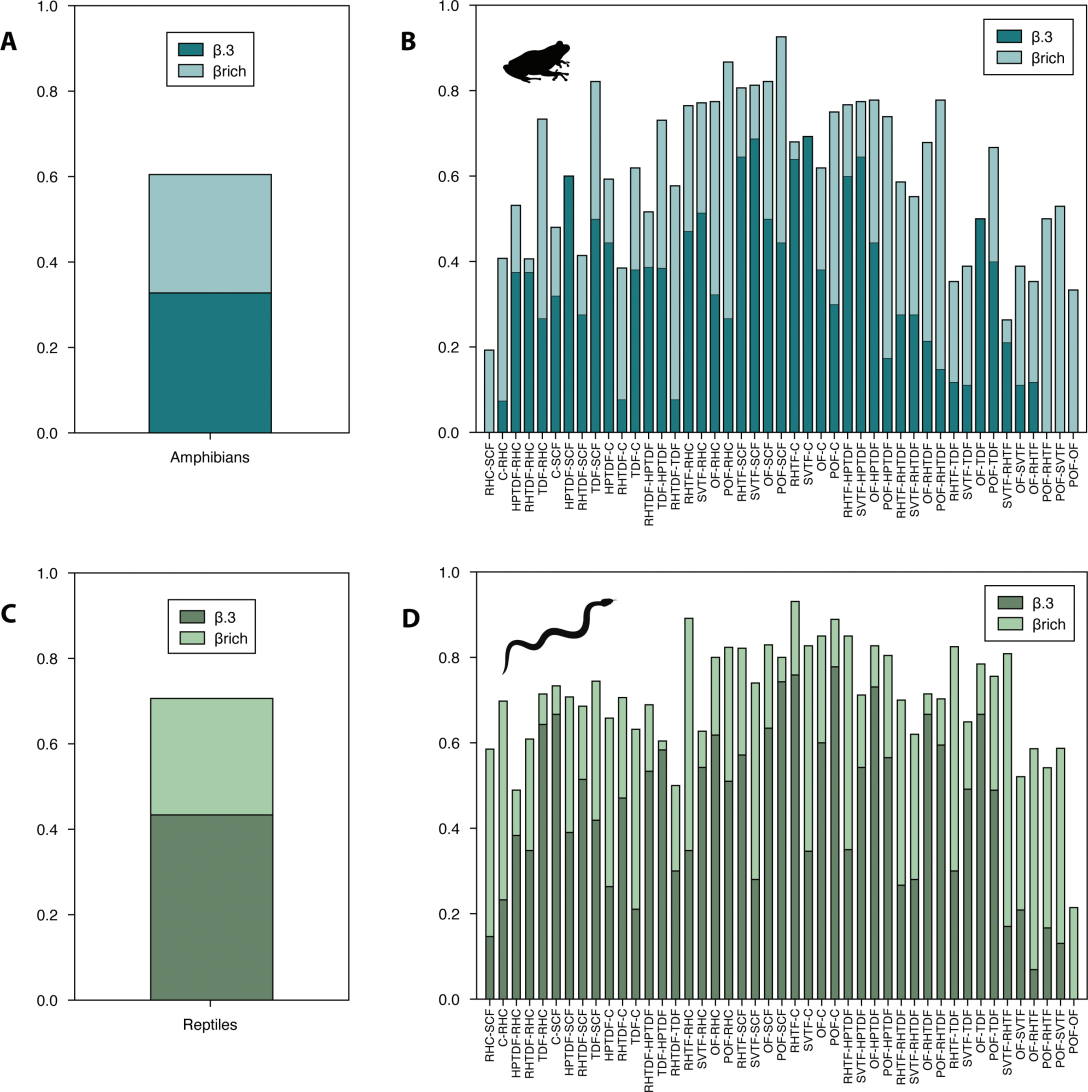
Taxonomic beta diversity of amphibians and reptiles considering the turnover (*β.3*) and differences in richness (*βrich*) components by land cover/use types **A** total and **B** paired beta diversity of amphibians; and **C** total and **D** paired beta diversity of reptiles. Codes: sugar cane field (SCF), riparian habitat surrounded by crops (RH-C), cornfield (C), highly perturbated tropical dry forest (HPTDF), tropical dry forest (TDF), riparian habitat surrounded by tropical forest (RH-TDF), riparian habitat surrounded temperate forest (RH-TF), secondary vegetation surrounded by temperate forest (SV-TF), oak forest (OF) and pine-oak forest (POF).

The taxonomic beta diversity of amphibians and reptiles was high overall in both groups, being slightly higher in reptiles (*βmulti* = 0.70) than in amphibians (*βmulti* = 0.60). The turnover component (*β.3* = 0.43 and *β.3* = 0.32, respectively) was the most significant contributor to taxonomic differentiation in all comparisons. In turn, the two groups had a low contribution from the differences in richness component (*βrich* = 0.27 for reptiles and *βrich* = 0.27 for amphibians) (Fig. 4A, C).

In contrast, amphibians and reptiles had divergent patterns in regard to beta diversity (both turnover and richness differences) in pairwise comparisons between land cover/use types. Only POF and OF showed differences in richness in amphibians and reptiles without the turnover component (Fig. 4B, D).

For amphibians, the pairwise comparisons with the highest beta taxonomic diversity were between POF and SCF (0.92), POF and RH-C (0.86), and TDF and SCF (0.82), while the lowest were between RH-C and SCF (0.19), SV-TF and RH-TF (0.26), and RH-TF and TDF (0.35). The comparisons with the highest turnover were between SV-TF and C (0.69), SV-TF and SCF (0.81), RH-TF and SCF, and SV-TF and HPTDF (0.77 respectively) (Fig. 4B). In these comparisons the component with the greatest contribution to differentiation was differences in richness in POF and RH-TDF (0.77), POF and RH-C (0.86), and POF and HPTDF (0.73). Some of the comparisons consisted only in the replacement component, like HPTDF and SCF (0.60), SV-TF and C (0.69), and OF and TDF (0.50). Likewise, RH-C and SCF (0.19), POF and OF (0.33), POF-SV-TF (0.52), and POF and RH-TDF (0.77) were only represented by differences in richness.

In relation to land cover/use types taxonomic beta diversity among reptiles was highest when comparing RH-TF and C (0.17), RH-TF and RH-C (0.54), and POF-C (0.11). The comparisons with the lowest values were POF-OF (0.21), HPTDF and RH-C (0.10), RH-TDF and TDF (0.20). The comparisons with the highest contribution of turnover component were between POF-C (0.11), RH-TF and C (0.17), and POF-SCF (0.05) comparisons, and for differences in richness component were between SV-TF and RH-TF (0.63), RH-TF and RH-C (0.54), and RH-TF-TDF (0.52). Similarly to amphibians, the comparison between POF-OF (0.21) was uniquely represented by differences in richness (Fig. 4D).

### ﻿Amphibian checklist and geographic expansion distributions

During nine years of observations, we recorded 20 species of amphibians belonging to 14 genera, nine families, and one order. The families with the highest species richness were Hylidae (seven species), Craugastoridae (three species) and Ranidae (three species). Ten of these species are endemic and under some protection category. Four species are under the Special Protection category, and one is under the Threatened category (SEMARNAT 2019). IUCN threat categories included two species that are considered Endangered and one, Vulnerable (UICN 2024). With respect to the Environmental Vulnerability Score (EVS), ten species are classified under low (L) vulnerability, seven species under medium (M), and one under high (H) vulnerability categories (Table 1).

**Table 1.** Checklist of amphibians and reptiles (August 2011 to December 2020) present in the various land/use types in a heterogenous landscape in west-central Mexico. Codes: sugar cane field (SCF), riparian habitat surrounded by crops (RH-C), cornfield (C), highly perturbed tropical dry forest (HPTDF), tropical dry forest (TDF), riparian habitat surrounded by tropical forest (RH-TDF), riparian habitat surrounded temperate forest (RH-TF), secondary vegetation surrounded by temperate forest (SV-TF), oak forest (OF), pine-oak forest (POF), endemic to Mexico (E), exotic (F), Special Protection (Pr), Threatened (A), Least Concern (LC), Near Threatened (NT), Vulnerable (VU), Endangered (EN), Low (L), Medium (M), High (H), records with distribution range extensions (*).

Taxonomic hierarchies and species	Common name	Endemism	Conservation status	SCF	RH-C	C	HPTDF	TDF	RH-TDF	RH-TF	SV-TF	OF	POF	Other land cover/use
**AMPHIBIA**														
**Order Anura**														
**Craugastoridae**														
*Craugastorcf.hobarthsmithi* (Taylor, 1937)	Smith’s pigmy tropical frog	E	EN, LC, L		X	X	X	X						
*Craugastoroccidentalis* (Taylor, 1941)	Taylor’s Barking Frog	E	LC, M			X		X	X	X	X	X	X	
*Craugastoraugusti* (Dugès, 1879)	Barking Frog		LC, L				X	X	X	X	X	X	X	
**Eleutherodactylidae**														
*Eleutherodactylus* sp.				X	X	X	X		X	X				
**Bufonidae**														
*Inciliusoccidentalis* (Camerano, 1879)	Pine Toad	E	LC, M								X	X	X	
*Rhinellahorribilis* (Wiegmann, 1833)	Giant Marine Toad		L	X		X	X		X					
**Hylidae**														
*Exerodontasmaragdina* (Taylor, 1940)	Emerald Tree Frog	E	Pr, LC, M								X			
*Dryophytesarenicolor* (Cope, 1866)	Canyon Tree Frog		LC, L					X	X	X	X			
*Dryophyteseximius* (Baird, 1854)	Mountain Tree Frog	E	LC, M						X		X			
Sarcohylahapsa* Campbell, Brodie, Caviedes-Solis, Nieto-Montes de Oca, Luja, Flores-Villela, Garcia-Vazquez, Sarker & Wostl, 2018	Northern Streamside Tree Frog	E	Pr, LC, L							X				
*Smiliscabaudinii* (Duméril & Bibron, 1841)	Common Mexican treefrog		LC, L											
*Smiliscafodiens* (Boulenger, 1882)	Lowland Burrowing Tree Frog		LC, L											
*Tlalocohylasmithii* (Boulenger, 1902)	Dwarf Mexican Tree Frog	E	LC, M											
**Leptodactylidae**														
*Leptodactylusmelanonotus* (Hallowell, 1861)	Black Jungle-Frog		LC. L	X	X				X					
**Microhylidae**														
*Hypopachusvariolous* (Cope, 1866)	Mexican Narrow-mouthed Toad		LC, L						X					
**Phyllomedusidae**														
*Agalychnisdacnicolor* (Cope, 1864)	Mexican Giant Tree Frog	E	LC, M		X									
**Ranidae**														
Ranacf.forreri (Boulenger, 1883)	Forrer’s leopard frog		Pr, LC, L						X					
*Rananeovolcanica* Hillis & Frost, 1985	Transverse Volcanic Leopard Frog	E	A, NT, M	X	X				X		X	X		
*Ranamegapoda* Taylor, 1942	Big-footed Leopard Frog	E	Pr, VU, H											X
**Scaphiopodidae**														
*Speamultiplicate* (Cope, 1863)	Mexican Spadefoot		LC, L				X							
**REPTILIA**														
**Orden Squamata**														
**Anguidae**														
*Elgariakingii* Gray, 1838	Arizona Alligator Lizard		Pr, LC, M				X			X		X	X	
**Anolidae**														
*Anolisnebulosus* (Wiegmann, 1834)	Clouded Anole	E	LC, M		X	X	X	X	X	X	X	X	X	
**Geckonidae**														
*Hemidactylusfrenatus* Duméril & Bibron, 1836	Asian House Gecko	F												X
**Iguanidae**														
*Ctenosaurapectinata* (Wiegmann, 1834)	Mexican Spinytail Iguana	E	A, H	X	X		X	X	X		X			
**Phrynosomatidae**														
*Sceloporusdugesii* Bocourt, 1874	Duges’ Spiny Lizard	E	LC, M			X	X							
*Sceloporusheterolepis* Boulenger, 1895	Dorsalkeel Spiny Lizard	E	LC, H					X	X	X	X	X	X	
*Sceloporushorridus* Wiegmann, 1834	Horrible Spiny Lizard		LC, M					X	X		X	X	X	
*Sceloporusnelson* Cochran, 1923	Nelson’s Spiny Lizard	E	LC, M				X							
*Sceloporustorquatus* Wiegmann, 1828	Torquate Lizard	E	LC, M					X						
*Sceloporusspinosus* Wiegmann, 1828	Eastern Spiny Lizard	E	LC, M						X					
*Sceloporusutiformis* Cope, 1864	Antesator	E	LC, H						X	X	X	X	X	
*Urosaurusbicarinatus* (Duméril, 1856)	Tropical tree lizard	E	LC, M	X			X	X						
**Phyllodactylidae**														
*Phyllodactyluslanei* Smith, 1935	Lane’s Leaf-toed Gecko	E	LC, H				X							
**Scincidae**														
*Plestiodondugesii* (Thominot, 1883)	Duges’ Skink	E	Pr, VU, H							X	X	X	X	
*Plestiodoncallicephalus* (Bocourt, 1879)	Mountain Skink		LC, M				X	X						
**Teiidae**														
*Aspidosceliscostatus* (Cope, 1878)	Western Mexico Whiptail	E	Pr	X	X	X	X							
*Aspidosceliscommunis* (Cope, 1878)	Colima Giant Whiptail	E	Pr, LC, M					X						
**Boidae**														
*Boasigma* (Smith, 1943)	Boa		A, LC, M				X	X						
**Colubridae**														
*Masticophismentovarius* (Duméril, Bibron & Duméril, 1854)	Neotropical Whip Snake	E	LC, L	X	X		X	X	X					
*Drymarchonmelanurus* (Duméril, Bibron & Duméril, 1854)	Blacktail Cribo		LC, L	X	X		X			X				
Lampropeltisruthveni* Blanchard, 1920	Ruthvens Kingsnake		A, NT, H		X		X				X		X	
*Leptophisdiplotropis* (Günther, 1872)	Pacific Coast Parrot Snake	E	A, LC, H					X						
*Masticophisbilineatus* (Jan, 1863)	Sonoran Whipsnake		LC, M			X								
*Oxybelisaeneus* (Wagler, 1824)	Mexican Vine Snake		L			X		X						
*Pituophisdeppei* (Duméril, 1853)	Mexican Bull Snake	E	A, LC, H				X	X						
*Senticolistriaspis* (Cope, 1866)	Green Rat Snake		LC, L	X			X							
*Sonoramutabilis* Stickel, 1943	Mexican Groundsnake	E	LC, H				X							
*Tantillabocourti* (Günther, 1895)	Bocourt’s Black-headed Snake	E	LC, L	X	X	X								
*Trimorphodontau* Cope, 1870	Mexican Lyre Snake	E	LC, M				X	X						
**Dipsadidae**														
*Diadophispunctatus* (Linnaeus, 1766)	Ring-necked snake		LC, L		X		X							
*Hypsiglenatorquata* (Günther, 1860)	Sinaloan Nightsnake		Pr, LC, L					X						
Imantodesgemmistratus* (Cope, 1861)	Central American Tree Snake		Pr, L				X							
*Leptodeiramaculata* (Hallowell, 1861)	Southwestern Cat-eyed Snake	E	Pr, LC, L								X			
*Leptodeirasplendida* Günther, 1885	Splendid Cat-eyed Snake	E	Pr, LC, H		X			X						
*Rhadinaea Hesperia* Bailey, 1940	Western Graceful Brown Snake	E	LC, M				X				X			
*Rhadinaeataeniata* (Peters, 1863)	Pine-Oak Snake	E	LC, M								X			
**Elapidae**														
Micrurusdistans* Kennicott, 1860	West Mexican Coral Snake	E	Pr, LC, H				X				X		X	
**Leptotyphlopidae**														
*Renahumilis* Baird & Girard, 1853	Western Blind Snake		LC, L				X	X						
**Natricidae**														
Thamnophiscopei* (Dugès, 1879)	Cope’s mountain meadow snake	E	Pr, VU, H				X							
*Storeriastorerioides* (Cope, 1866)	Mexican Brown Snake	E	LC, M								X	X	X	
*Thamnophiseques* (Reuss, 1834)	Mexican Garter Snake		A		X				X					
*Thamnophismelanogaster* (Peters, 1864)	Blackbelly Garter Snake	E	A, LC, L											X
*Thamnophiscyrtopsis* (Kennicott, 1860)	Black-necked Garter Snake		A, LC, L				X				X		X	
**Typhlopidae**														
*Indotyphlopsbraminus* (Daudin, 1803)	Bootlace Snake	F			X	X		X						
**Viperidae**														
*Agkistrodonbilineatus* Günther, 1863	Cantil Viper		Pr, NT, M		X	X		X						
*Crotalus Basiliscus* (Cope, 1864)	Basilisk Rattlesnake	E	Pr, LC, H	X	X					X				
*Crotalustriseriatus* Wagler, 1830	Western Dusky Rattlesnake	E	H										X	
**Order Testudines**														
**Kinosternidae**														
*Kinosternonintegrum* Le Conte, 1854	Mexican Mud Turtle	E	Pr, M	X	X						X			

We documented extensions in the known distribution range of *Sarcohylahapsa* (Suppl.material 1: fig. S1A). *Sarcohylahapsa* is endemic to western Mexico (Campbell et al. 2018). It was observed in September 2011 in the RH-TF at 1842 m a.s.l. (20°38,58'N, 104°3,14'W). This species was recently split from the widespread Mexican hylid *Sarcohylabistincta* (Campbell et al. 2018). This extends the known distribution by 38 km from its closest record, base La Ciénega, Sierra de Quila (Rosas-Espinoza et al. 2013; GBIF 2023a) (Suppl.material 1: fig. S2).

### ﻿Reptile checklist and geographic expansion distributions

We recorded 48 species of reptiles belonging to 34 genera, 17 families, and two orders (Table 1). The families with the highest species richness were Colubridae (11 species), Phrynosomatidae (eight species), Dipsadidae (seven species) and Natricidae (five species). More than half of these species are endemic to Mexico (62.5%). All the species are native to Mexico except *Hemidactylusfrenatus* and *Indotyphlopsbraminus*, which are native to the Eastern Hemisphere. According to the Mexican species protection list, 21 species are in a category of risk, of which 13 are under Special protection and eight are Threatened (SEMARNAT 2019). IUCN threat categories classify two species as Endangered, one as Vulnerable, and two as Near Threatened (UICN 2024). According to the EVS (Wilson et al. 2013a), 12 species are under low (L) vulnerability, 18 species under medium (M), and 14 are below the high (H) vulnerability categories (Table 1).

We documented extensions in the known distribution range of three species of reptiles. *Lampropeltisruthveni* was observed in September 2011 in SV-TF (20°40'01"N, 103°52'23"W) (Suppl.material 1: fig. S1B). There are a few records of this species in the Trans-Mexican Volcanic Belt. Its distribution is extended by at least 44 km from its closest known record near Sierra de Quila, Jalisco (GBIF 2023b) (Suppl.material 1: fig. S3A).

*Thamnophiscopei* is endemic to Mexico (Suppl.material 1: fig. S1C). It was observed in September 2012 at HPTDF (20°41,52'N, 103°49,35'W). This extends the known distribution range of the species by 39 km from La Quemada, Jalisco (GBIF 2023c) (Suppl.material 1: fig. S3B).

*Imantodesgemmistratus* was observed in September 2012 in HPTDF (20°41’,41"N, 103°50'33"W) (Suppl.material 1: fig. S1D). This extends its known distribution by 36 km from 2.3 km presa El Texcalame, (GBIF 2023d) (Suppl.material 1: fig. S3C).

## ﻿Discussion

We achieved high sampling efforts for both amphibians and reptiles at all sites, so the samples can be considered representative of both groups. Support for this assessment comes also from having recorded the same number of species of amphibians in another seven years of non-systematic surveys and species observations within the study area. In contrast, the number of species of reptiles increased from 39 to 48 species, the earlier systematic survey (August 2011 to September 2013) detected 81% of the reptile species present there.

### ﻿Alpha taxonomic diversity

Monitoring taxonomic diversity has been proposed as a tool to develop ecosystem management plans, ecological restoration projects, and the creation of protected areas (Somerfield et al. 2008), and guide conservation strategies in areas where biodiversity loss is occurring at an accelerated rate (Hernández-Salinas et al. 2023). In the present work, we obtained sound estimates of the alpha taxonomic diversity of amphibians and reptiles in the different land cover/use types. This may have been helped by the fact that the study area consists of a heterogeneous landscape with different land cover types with temperate and tropical native vegetation. Although, the HPTDF had a high level of disturbance, it is known that secondary TDF in human-dominated landscapes can support substantial amphibian diversity (Suazo-Ortuño et al. 2015). Moreover, animals can move between patches with native vegetation and crops to seek shelter (when there is vegetation cover), food, or to reproduce (during the rainy season). Iglesias-Carrasco et al. (2023) reported that monocultures and poly-specific plantations affect the conservation and ecological value of these habitats to both amphibian and reptile communities detrimentally and alter the evolutionary processes shaping these communities. In contrast, forests with lower impact disturbances can, to some extent, serve as reservoirs of species. Another important factor to consider is the resilience of species to disturbance, since some species populations do not seem to be affected as markedly as others.

Only the RH-C (five species) had higher taxonomic distinctiveness of amphibians than expected from the model. RH-TDF (ten species) and SCF (four species) had the highest alpha taxonomy diversity within the model. TDF did not have the highest alpha taxonomic richness against what we expected, even though it is recognized as a neotropical ecosystem with an important amphibian richness (23% of Mexican amphibians) (Ceballos and García 1995). Suazo-Ortuño et al. (2011) and Álvarez-Grzybowska et al. (2020) reported that TDF in western Mexico sheltered a high amphibian richness but only during the rainy season. Hromada et al. (2021) highlighted the importance of water for amphibians. Even when water quality was reduced, they found that amphibian diversity was higher in ponds surrounded by low-intensity agricultural areas influenced by the surrounding forest and pasture. Our alpha taxonomic results for amphibians are consistent with the observation that amphibians are highly associated with water (Wells 2007). Additionally, it has been reported that diversity is higher in tropical ecosystems compared to temperate ones. The high taxonomic diversity in the SCF may be assisted by its proximity to the RH-TDF and the fact that amphibians can move between land cover/use types, especially during the rainy season.

Almost all the land cover/use types used by reptiles had a higher alpha taxonomic distinctiveness than the average within the model. These results suggest that reptiles can maintain high alpha taxonomic diversity even in heterogeneous landscapes. SCF (nine species), RH-C (14 species), and RH-TF (seven species) had the highest alpha taxonomic diversity within the model, and RH-TDF (eight species) and HPTDF medium ones (24 species). Agricultural systems can vary greatly in structure; uniform agroecosystems like monocultures exhibit shallow levels of biodiversity (Altieri and Nicholls 2004), while more complex agroecosystems shelter high biodiversity (Isbell et al. 2017). However, in a landscape context, certain cultivated areas can function as buffer zones at natural edges (Gascon et al. 2004) or as corridors between native habitat fragments (García et al. 2006). SCF had high alpha taxonomic diversity probably because this crop provides continuous cover for years (approximately six years) and shelters a high population of rodents and other prey such as lizards, frogs, and toads that can serve as food for snakes. RH-C, RH-TF, and RH-TDF also provided habitat, water, and food availability for different species. It has been reported that TDF shelters 34% of Mexican reptiles (Ceballos and García 1995), and even secondary TDF harbors high reptile’ diversity (Suazo-Ortuño et al. 2015), as we found in HPTDF.

### ﻿Beta taxonomic diversity

Factors that contribute to different components of beta diversity in amphibians and reptiles include the physiological limits of the species (*βrich*) and speciation processes (*β.3*), especially in taxa with low mobility (Baselga et al. 2012). The heterogeneous mosaic arrangement with patches of arable areas, forest remnants, and the temperate-tropical configuration in the study area helps explain high beta diversity values for both groups (Ceballos and García 1995; Baselga 2010). Additionally, elevation has been considered a strong promoter of beta taxonomic diversity because it promotes contrasting characteristics within the gradient of vegetation types that change with elevation (Baselga et al. 2012; Ochoa-Ochoa et al. 2014).

For the region evaluated, the turnover component (*β.3*) contributed the most to taxonomic differentiation because of the configuration with abrupt changes in patches mentioned earlier. Because of the narrow distribution ranges of amphibians and reptiles, this is consistent with previous work (Baselga et al. 2012; Ochoa-Ochoa et al. 2014; Rodríguez et al. 2019). However, it has been observed that differences in richness component (*βrich*) can be major in groups with low mobility not only with respect to nesting, but also gain or loss of species among the sites evaluated (Carvalho et al. 2012; Calderón-Patrón et al. 2016), as was the case with amphibians where both components contributed almost equally to diversity of taxonomic differentiation.

Taxonomic beta diversity can also be expected to differ between groups with different evolutionary histories; notably, it has been reported that amphibians show higher taxonomic beta diversity due to dispersal limitations and their dependence on water bodies (Ochoa-Ochoa et al. 2014; Calderón-Patrón et al. 2016). However, we observed the opposite pattern, with reptiles showing the highest taxonomic beta diversity of the two groups. This could be due to the study area being a highly heterogeneous region with disturbed areas promoting greater spatial differentiation for reptiles.

The relationship between alpha and beta taxonomic diversity remains poorly understood (Ochoa-Ochoa et al. 2014). In the present study, the reptiles had the highest species richness and beta diversity. However, amphibians showed the highest average taxonomic distinctiveness (at the 90% level) because of the inclusion of members of different orders and families. In contrast, the supra-specific levels of reptiles differed at a 70% level at the genus and family level.

Although paired comparisons between the two groups reveal differences in beta diversity, we also documented common responses. In amphibians, the highest beta diversity occurred between conserved (POF, TDF) and disturbed (SCF, RHC) habitats. This reflects a high sensitivity to local disturbance, especially considering that this group was strongly influenced by the differences in richness component of beta diversity, as in the case of the comparison of POF with RH-C (one of the comparisons with the highest beta diversity). This indicates that despite shared taxa, supra-specific levels are aggregated to cause differences in richness between the paired comparisons. In the same way, in reptiles, we found that the comparisons with the highest beta diversity occurred between conserved (RH-TF, POF) and disturbed (C, RH-C) habitats, showing a consistent pattern with a predominance of the turnover component. This resulted from changes in the taxa set between these habitats. These results suggest that disturbance could become more important for taxonomic beta diversity of amphibians and reptiles than temperate *vs.* tropical conditions, given its high potential to threaten species and populations, acting more aggressively than the evolutionary history of species (Ochoa-Ochoa et al. 2014).

### ﻿Amphibians and Reptiles checklist and geographic range extensions

Our work found that the study area shelters 40.4% and 28.1% of Jalisco’s state amphibians (52 species) and reptiles (171 species), respectively (Cruz-Sáenz et al. 2017). This finding highlights the region’s significance for conserving amphibians and reptiles. The 68 species (20 and 48 amphibians and reptiles, respectively) observed in this study were higher than in nearby natural areas with similar land cover types, for example La Primavera Forest (with the presence of POF, OF, RH, and TDF) with 56 species (17 amphibians and 39 reptiles) (Reyna-Bustos et al. 2007) and Tequila Volcano (pine forest, POF, OF, RH, TDF, Mountain cloud forest, grassland) with 32 species (10 amphibians and 22 reptiles) (Rojo-Guti érrez et al. 2022). Conversely, the species richness was comparable to that of Sierra de Quila (oak pine forest, OF, RH, and TDF), with 69 species (23 amphibians and 46 reptiles) (Santiago-Pérez et al. 2012). Salcido-Rodríguez et al. (2023) reported 18 amphibians and 47 reptile species (65 species total) for Sierra de Tesistán with land cover types POF, OF, and secondary vegetation in the ecotone between OF and TDF. Finally, Flores-Covarrubias et al. (2012) reported 20 amphibians and 40 reptiles (61 species) for the TDF, Thorn scrub, OF, POF, grassland, secondary vegetation, and agricultural lands in Hostotipaquillo. The diversity of amphibians and reptiles observed in this study underlines the richness of the herpetofauna in the area, even though it is a heterogeneous landscape with crops.

Many of the recorded species (56.5%) in this heterogeneous landscape are endemic to Mexico. This was true for more than half of the species of reptiles, highlighting the area’s role in conserving existing native biodiversity. This could be due to its geographical position and the influence of the Trans-Mexican Volcanic Belt which, through the complexity of the landscape, promotes processes of speciation and endemism and is considered one of the most diverse zones in the country (Flores-Villela 1993). We observed two exotic species in the study area: the snake *I.braminus* and the lizard *H.frenatus*. According to CONABIO (2020), exotic species are Mexico’s third most significant threat to biodiversity. The dangers associated with their presence in the study area remain unknown (Farr 2011). Also, according to NOM-059 (2019), IUCN (2024) and Wilson (2013a, 2013b), 73% of the species (11 amphibians and 39 reptiles) are under some protection status, highlighting the importance of the existing matrix of different habitats (especially different native vegetation types) to shelter endemic amphibians and reptiles or those with conservation concerns.

Knowing the local distribution of species is essential to monitor and manage local wildlife (Pisanty et al. 2016) as well as the first step in biodiversity conservation. In this study, we found species with a wide distribution in Mexico (e.g., *A.nebulosus*, *Pituophisdeppei*, and *Kinosternonintegrum*) or in west-central Mexico (e.g., *Phyllodactyluslanei*, *Sceloporusdugesii*, and *Sonoramutabilis*), as well as others with very restricted ones, like *S.hapsa*, *L.ruthveni*, and *T.copei*. Extensions in the distribution range of one amphibian and three reptile species shows that Mexico’s west-central region has not been studied enough towards the mountain areas within the Trans-Mexican Volcanic Belt and the Sierra Madre Occidental.

## ﻿Conclusions

We found that the amphibian and reptile taxonomic diversity in the studied landscape results from i) remnants of native vegetation, even with some level of disturbance, ii) the existence of a heterogeneous matrix with different land cover/use types, albeit with a higher number of land cover types, iii) availability of water during the whole year in some of the land cover types, iv) connectivity between areas allowing the animals to move between different land cover/use types. Finally, the proximity of the study area to mountainous areas like La Primavera or Tequila Volcano is probably another factor to consider. These mountains are natural areas that harbor wildlife and that might act as species pools that could disperse to the study area.

## ﻿Recommendations

Knowing the distribution of species at different spatial scales, having complete checklists, and analyzing diversity in its various facets at both alpha and beta levels are essentials for species management and conservation. Variation in alpha and beta taxonomic diversity presents a challenge for conservation strategies and management plans as they need to consider differences between sites. It is vital to consider endemic species, particularly those under conservation categories or those associated with native vegetation cover types with low disturbance, so management practices can encourage their presence and abundance. In this sense, preserving remaining natural forests and those with different levels of disturbance is necessary for conserving amphibian and reptile communities. Moreover, this study highlights the need for specific conservation strategies and recommendations to be integrated into broader landscape-level conservation planning. Nowadays, the great rate of land cover changes highlights the need to promote the existence of a connected heterogeneous landscape with different land cover/use types, thus enhancing the ability to conserve amphibians and reptiles. The other patches can offer shelter, water, and food permanently or temporarily. Even more, encouraging the connection among different land cover/use types will ensure that amphibians and reptiles can move between patches of this matrix.

## ﻿Additional information

### Author ORCIDs

Verónica Carolina Rosas-Espinoza https://orcid.org/0000-0001-8595-3203

Eliza Álvarez-Grzybowska https://orcid.org/0000-0003-3580-3869

Arquímedes Alfredo Godoy González https://orcid.org/0000-0002-6313-0238

Ana Luisa Santiago-Pérez https://orcid.org/0000-0001-7494-9129

Karen Elizabeth Peña-Joya https://orcid.org/0000-0002-7237-5894

Fabián Alejandro Rodríguez-Zaragoza https://orcid.org/0000-0002-0066-4275

Leopoldo Díaz Pérez https://orcid.org/0000-0003-0271-9257

Francisco Martín Huerta Martínez https://orcid.org/0000-0001-6923-3425

### Data availability

All of the data that support the findings of this study are available in the main text or Supplementary Information.

## Supplementary materials

Supplementary material 1 Supplementary information Data type docx Explanation note **table S1.** Sample-based rarefaction curves generated using presence-absence data for both amphibians and reptiles. We report observed and expected species by land cover/use type using non-parametric estimators. Codes: sugar cane field (SCF), riparian habitat surrounded by crops (RH-C), cornfield (C), highly perturbated tropical dry forest (HPTDF), tropical dry forest (TDF), riparian habitat surrounded by tropical forest (RH-TDF), riparian habitat surrounded temperate forest (RH-TF), secondary vegetation surrounded by temperate forest (SV-TF), oak forest (OF), pine-oak forest (POF) and number of species observed (Sobs). **fig. S1.** Species with the documented range extensions. **A***Sarcohylahapsa***B***Lampropeltisruthveni***C***Thamnophiscopei***D***Imantodesgemmistratus* in a heterogeneous landscape in west-central Mexico. All photos by Eliza Álvarez-Grzybowska, except B) by Aldo Dávalos Martínez. **fig. S2.** Records of *Sarcohylahapsa* in Jalisco state (black triangles, GBIF 2023a). The red star shows our record of range extension for *S.hapsa*. **fig. S3.** Records of **A***Lampropeltisruthveni***B***Thamnophiscopei*, and **C***Imantodesgemmistratus* in Jalisco state (black triangles, GBIF 2023). The red stars show our records of range extension for these different species. The colored areas are published distributions (IUCN 2024). https://binary.pensoft.net/file/1126423This dataset is made available under the Open Database License (http://opendatacommons.org/licenses/odbl/1.0/). The Open Database License (ODbL) is a license agreement intended to allow users to freely share, modify, and use this Dataset while maintaining this same freedom for others, provided that the original source and author(s) are credited.Verónica Carolina Rosas-Espinoza, Eliza Álvarez-Grzybowska, Arquímedes Alfredo Godoy González, Ana Luisa Santiago-Pérez, Karen Elizabeth Peña-Joya, Fabián Alejandro Rodríguez-Zaragoza, Leopoldo Díaz Pérez, Francisco Martín Huerta Martínez
