# Supplementary material for: ﻿Taxonomic diversity of amphibians (Amphibia, Anura) and reptiles (Reptilia, Testudines, Squamata) in a heterogeneous landscape in west-central Mexico: a checklist and notes on geographical distributions
Source: Zookeys. 2024 Sep 2;1211:29–55. doi: 10.3897/zookeys.1211.122565 (PMC11384138; doi:10.3897/zookeys.1211.122565)
Supplement: Supplementary material 1 — Supplementary information [file zookeys-1211-029_article-122565__-s001.docx]

**Supplementary materials**

**Taxonomic diversity of amphibians (Amphibia, Anura) and reptiles (Reptilia, Testudines, Squamata) in a heterogeneous landscape in west-central Mexico: a checklist and notes on their geographical distributions**

Verónica Carolina Rosas-Espinoza^1^, Eliza Álvarez-Grzybowska^1^, Arquímedes Alfredo Godoy González^1^, Ana Luisa Santiago-Pérez^2^, Karen Elizabeth Peña-Joya^3^, Fabián Alejandro Rodríguez-Zaragoza^1^, Leopoldo Díaz Pérez^1^ and Francisco Martín Huerta Martínez*^4^

^1^Laboratorio de Ecología Molecular, Microbiología y Taxonomía (LEMITAX), Departamento de Ecología, Centro Universitario de Ciencias Biológicas y Agropecuarias, Universidad de Guadalajara, Camino Ramón Padilla Sánchez 2100, CP 45200, Zapopan, Jalisco, México

^2^Departamento de Producción Forestal, Centro Universitario de Ciencias Biológicas y Agropecuarias, Universidad de Guadalajara, Camino Ramón Padilla Sánchez 2100, CP 45200, Zapopan, Jalisco, México

^3^Laboratorio de Ecología, Paisaje y Sociedad, Centro Universitario de la Costa, Universidad de Guadalajara, Puerto Vallarta 48280, Jalisco, México

^4^ Centro de Estudios en Interacciones Ecológicas, Departamento de Ecología, Centro Universitario de Ciencias Biológicas y Agropecuarias, Universidad de Guadalajara, Camino Ramón Padilla Sánchez 2100, CP 45200, Zapopan, Jalisco, México

^*^Corresponding author: Martín Huerta Martínez ([martin.huerta@academicos.udg.mx](mailto:martin.huerta@academicos.udg.mx))

**Supplementary Table S1.** Sample-based rarefaction curves generated using presence-absence data for both amphibians and reptiles. We report observed and expected species by land cover/use type using non-parametric estimators. Codes: sugar cane field (SCF), riparian habitat surrounded by crops (RH-C), cornfield (C), highly perturbated tropical dry forest (HPTDF), tropical dry forest (TDF), riparian habitat surrounded by tropical forest (RH-TDF), riparian habitat surrounded temperate forest (RH-TF), secondary vegetation surrounded by temperate forest (SV-TF), oak forest (OF), pine-oak forest (POF) and number of species observed (Sobs).

| Land cover/use type | Sobs | Chao 2 | Jackknife 1 | Jackknife 2 | Average |
| --- | --- | --- | --- | --- | --- |
| Amphibians |  |  |  |  |  |
| SCF | 7 | 7.22 | 8.78 | 8.29 | 8.10 |
| RH-C | 9 | 9.67 | 11.67 | 11.96 | 11.10 |
| C | 6 | 6.67 | 8.67 | 8.96 | 8.10 |
| HPTDF | 8 | 8.67 | 10.67 | 10.96 | 10.10 |
| TDF | 5 | 6.31 | 7.63 | 9.23 | 7.72 |
| RH-TDF | 10 | 11.05 | 13.5 | 13.93 | 12.83 |
| RH-TF | 6 | 6 | 6.88 | 6 | 6.29 |
| SV-TF | 7 | 7 | 7.88 | 7 | 7.19 |
| OF | 5 | 7.63 | 7.63 | 9.88 | 8.38 |
| POF | 3 | 3 | 3.88 | 4.63 | 3.84 |
| Reptiles |  |  |  |  |  |
| SCF | 7 | 7 | 7 | 7 | 7 |
| RH-C | 13 | 21.89 | 17.44 | 21.33 | 20.22 |
| C | 7 | 9.67 | 9.67 | 12 | 10.45 |
| HPTDF | 12 | 14.96 | 16.44 | 18.97 | 16.79 |
| TDF | 14 | 24.5 | 21.88 | 14 | 20.13 |
| RH-TDF | 10 | 11.31 | 13.5 | 14.57 | 13.13 |
| RH-TF | 5 | 5 | 5.88 | 5.34 | 5.41 |
| SV-TF | 18 | 22.08 | 25 | 27.79 | 24.96 |
| OF | 12 | 12 | 12.88 | 11.7 | 12.29 |
| POF | 9 | 9.29 | 10.75 | 10.96 | 10.33 |

**
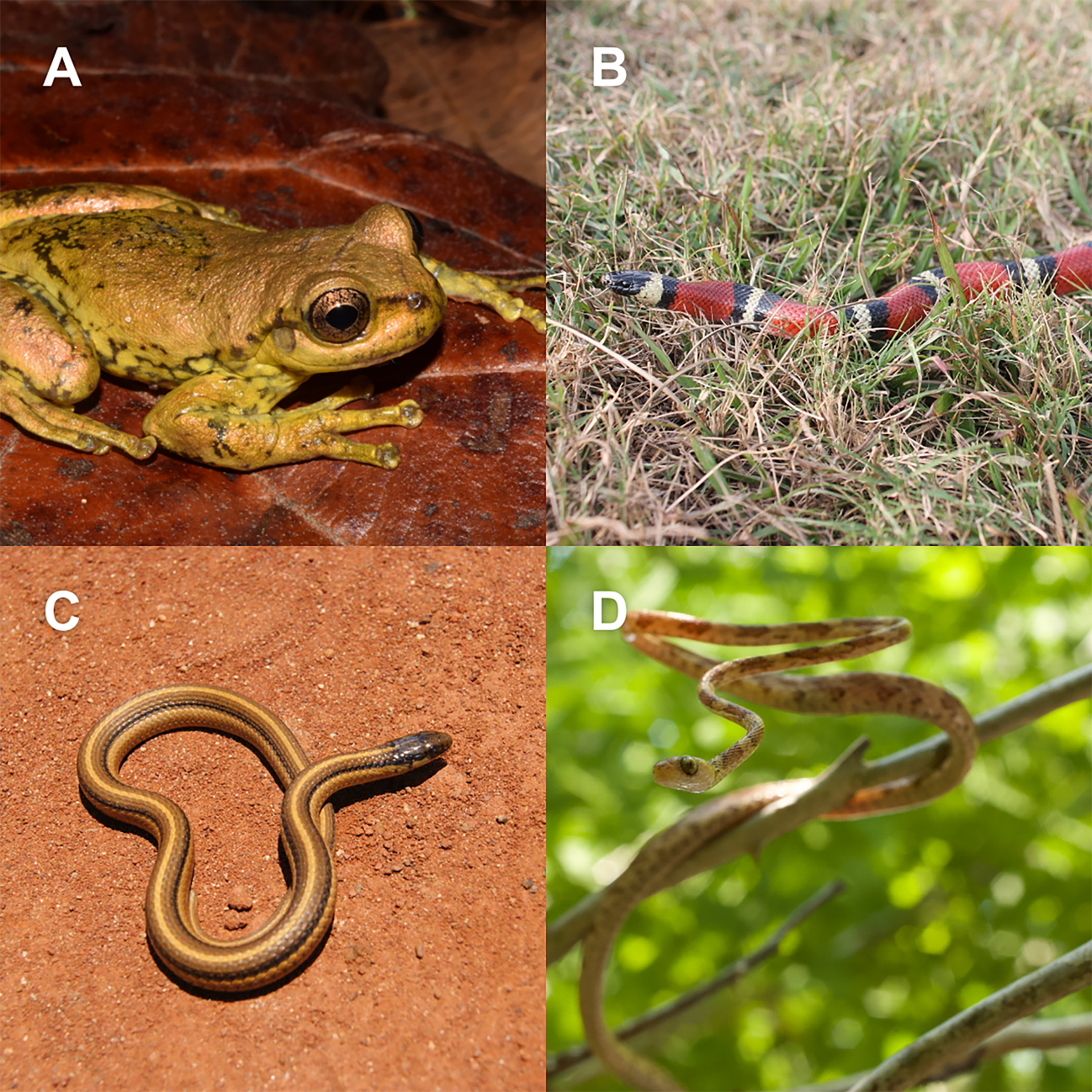
**

**Supplementary Figure S1.** Species with the documented range extensions. A) *Sarcohyla hapsa*, B) *Lampropeltis ruthveni*, C) *Thamnophis copei,* D) *Imantodes gemmistratus* in a heterogeneous landscape in west-central Mexico. All photos by Eliza Álvarez-Grzybowska, except B) by Aldo Dávalos Martínez.


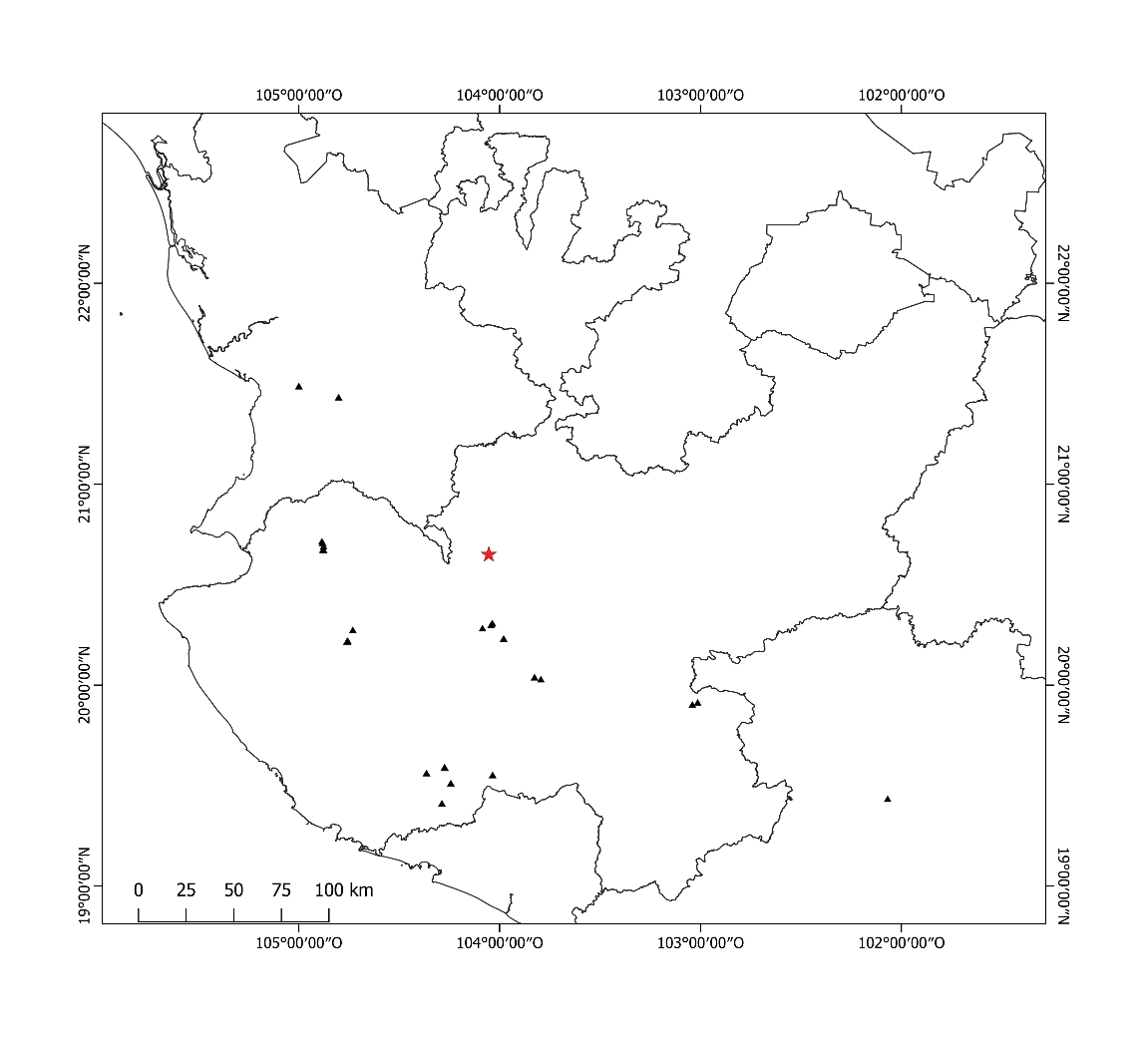


**Supplementary Figure S2.** Records of *Sarchohyla hapsa* in Jalisco state (black triangles, GBIF 2023a). The red star shows our record of range extension for *S. hapsa*.

|  |
| --- |

| 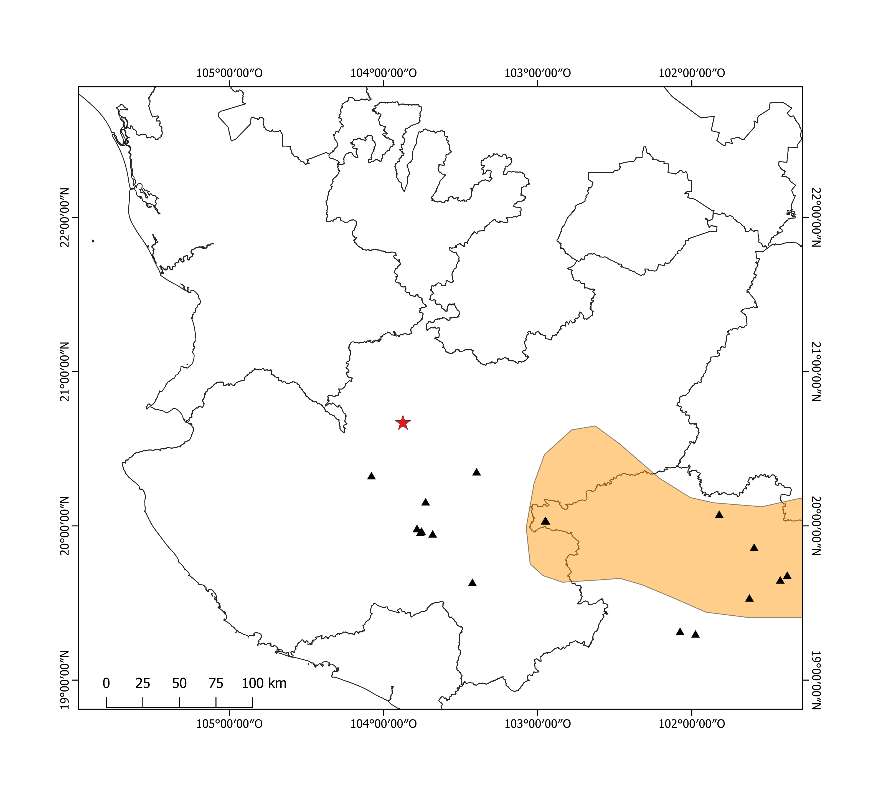  A | 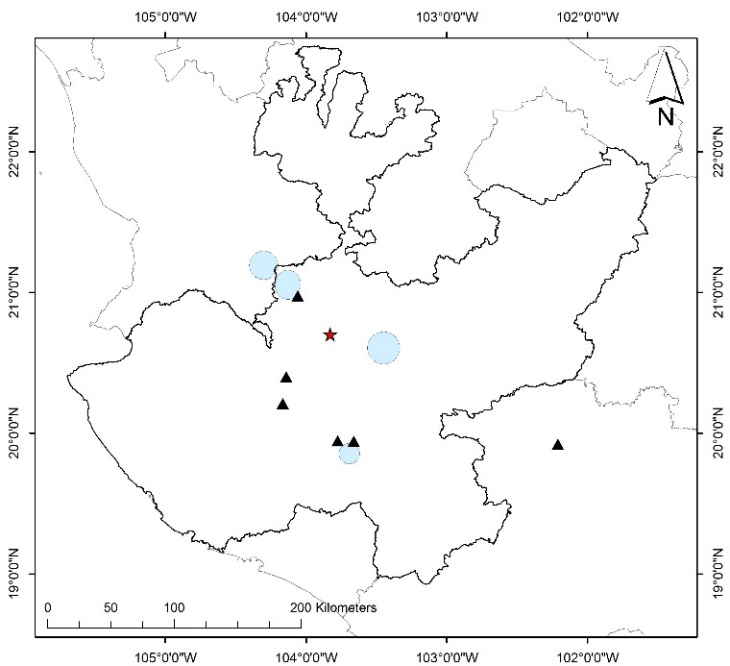  B |
| --- | --- |
| 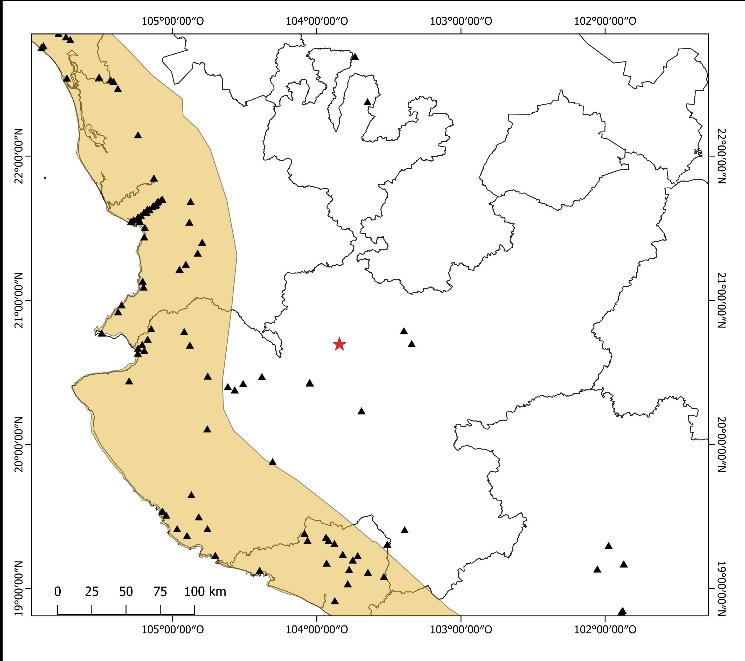  C |  |

**Supplementary Figure S3.** Records of A) *Lampropeltis ruthveni*, B) *Thamnophis copei*, and C) *Imantodes gemmistratus* in Jalisco state (black triangles, GBIF 2023). The red stars show our records of range extension for these different species. The colored areas are published distributions (IUCN 2024).
